# The Role of Coronary Physiology Assessment in the Diagnosis and Treatment of Stable Angina. Dive Inside Recent Findings of Diffuse Coronary Disease Treatment

**DOI:** 10.31083/j.rcm2503108

**Published:** 2024-03-15

**Authors:** Valentin Chioncel, Flavius-Alexandru Gherasie

**Affiliations:** ^1^Department of Cardiology, University of Medicine and Pharmacy “Carol Davila”, 050474 Bucharest, Romania; ^2^Emergency Clinical Hospital Dr. Bagdasar-Arseni, 041915 Bucharest, Romania

**Keywords:** coronary physiology, coronary artery disease, angina, percutaneous coronary intervention, coronary microcirculation dysfunction, fractional flow reserve, hyperemic pullback pressure gradient

## Abstract

Coronary physiology is widely used to assess epicardial coronary lesions in patients with stable angina. Based on the available evidence, physiology plays a crucial role in diagnosing and treating patients. There have been invasive methods for determining cardiac physiology, such as fractional flow reserve and instantaneous wave-free ratio. Still, new non-invasive approaches provide extra anatomical information, such as fractional flow reserve computed tomography (FFR-CT) based on computed tomography and physiology based on angiography. Even though FFR-guided percutaneous coronary intervention (PCI) is clinically beneficial, one-third of patients retain suboptimal FFR after the procedure, associated with severe adverse events, rendering PCI in diffuse coronary artery disease questionable. Using the pullback pressure gradient (PPG), we can analyze the magnitude and extent of pressure losses; a lower value may indicate diffuse disease, while a high value with an abrupt curve may indicate focal disease. Since PCI is not the best option for treating diffuse coronary disease, current strategies focus on conservatively using medical therapy or bypass surgery. It has been demonstrated that patients with diffuse disease of the left anterior descending (LAD) are at a greater risk of developing occlusion of the left internal mammary artery graft than those with focal disease and that maximal medical therapy may be the most effective treatment for these patients.

## 1. Introduction

Ischemic heart disease is a leading factor of morbidity and mortality across the globe, and angina is the most prevalent symptom. A comprehensive history and examination are essential to differentiate between these causes and recognize patients suffering from acute coronary syndrome. Coronary artery disease (CAD) is characterized by atherosclerosis developing in the epicardial vessels, which may be obstructive or non-obstructive.

Several basic tests can be completed in patients with suspected CAD, such as bio-chemical testing, a resting electrocardiogram, resting echocardiography, and, in selected cases, ambulatory electrocardiogram (ECG) monitoring. To estimate obstructive CAD’s pre-test probability (PTP), it is necessary to consider factors such as age, gender, and the nature of symptoms. Over time, PTP received an update due to new data from the studies. Using data obtained from the PROMISE trial (Prospective Multicenter Imaging Study for Evaluation of Chest Pain), 50% of patients initially categorized as intermediately likely to have obstructive CAD were revised to a PTP of 15% [1]. It has been shown that the results in patients classified with the new PTP 15% are reliable (the annual risk of cardiovascular death or myocardial infarction is 1%) [2]. In patients whose chronic conditions and overall quality of life make revascularization inappropriate, CAD can be diagnosed clinically, and only medical treatment is necessary. If the diagnosis of CAD is uncertain, it may be appropriate to conduct non-invasive functional tests for myocardial ischemia, including stress cardiac magnetic resonance (CMR), stress echocardiography, perfusion changes by single-photon emission computed tomography (SPECT), positron emission tomography (PET), myocardial contrast echocardiography, or contrast cardiovascular magnetic resonance (CMR). An ischemic condition may be induced by exercise or pharmacological agents, either by increasing the load on the heart or by oxygen demand by vasodilators.

It is reasonable to perform directly invasive coronary angiography (ICA) when a patient’s clinical probability of coronary artery disease is high, the symptoms are unresponsive to medical treatment, or the angina is typical when performing physical activity at a low level. Additionally, non-invasive diagnostic tests may be recommended to determine the diagnosis and assess the risk of an event in patients whose clinical assessment cannot exclude CAD. According to the current guidelines, the preliminary test for CAD diagnosis should be non-invasive functional ischemia imaging or anatomical assessment using coronary CT angiography (CTA).

Optimal Medical Therapy (OMT) is the primary treatment method to manage CAD symptoms and prevent major cardiac events. However, additional revascularization procedures such as percutaneous coronary intervention (PCI) or coronary artery bypass graft surgery (CABG) can significantly increase quality of life and life expectancy. Invasive coronary angiography identifies significant CAD. Nevertheless, the relationship between stenosis severity, blood flow, and outcome is complicated, and the correspondence between a lesion’s visual evaluation and its physiological significance is inadequate [1].

For this reason, several tests can be used to determine blood flow in the coronary arteries through exercise or pharmacological provocation. Fractional flow reserve (FFR), invasive coronary physiology measurements with coronary guidewires with a pressure sensor, is routinely used as part of catheterization lab procedures [2, 3].

FFR refers to the ratio of the measured pressure distal of a coronary stenosis (Pd) compared to the pressure proximal to the stenosis, usually aortic pressure (Pa). Its original definition referred to the ratio of the maximal flow before and after stenosis. Nonetheless, pressure measurements are more straightforward and show a near-linear correlation with blood flow. The linear correlation between pressure and flow is only accurate when pressure measurements are conducted when the coronary resistance is at a minimum. The best way to minimize this resistance is through hyperemia. The most commonly used drug to induce hyperemia is adenosine, administered as a continuous intravenous infusion at a rate of 140 µg/kg/min or through an intracoronary bolus.

In clinical decision-making, an FFR value of 0.80 or lower indicates the need for revascularization, while a value above 0.80 suggests a conservative approach [4].

In the current European Society of Cardiology (ESC) guideline, FFR has a class 1A recommendation for identifying hemodynamically relevant coronary lesions in stable patients when evidence of ischemia is unavailable.

## 2. Understanding the Pathophysiology of Coronary Artery Disease

The coronary vasculature may be divided into two components: the epicardial coronary arteries, which arise from the aorta and supply the myocardium with oxygenated blood, and the coronary microvascular compartment [5]. It is important to emphasize that the macroscopic compartment estimated by ICA represents less than 10% of the coronary vasculature with a conductance function constituted by epicardial arteries (>400 µm). Microvascular compartments are responsible for 90% of coronary vasculature and consist of pre-arterioles (100 to 400 µm), arterioles (40 to 100 µm), and capillaries (<10 µm). To respond to tissue metabolism demands, pre-arterioles and arterioles regulate and distribute blood flow with maximum resistance to coronary flow.

In obstructive epicardial atherosclerosis, arterial tone maintains coronary blood flow, thereby reducing ischemia. Even though ICA is not capable of measuring coronary microcirculation, the clinical manifestation of coronary disease is dependent on both compartments being involved, perhaps simultaneously. Therefore, ICAs with coronary physiological indexes have a more significant clinical relevance as they can evaluate the entire coronary tree [6]. Patients with obstructive coronary artery disease have epicardial atherosclerotic lesions that may result in ischemia due to increased oxygen demands.

### INOCA: Diagnostic and Treatment

Ischemia associated with non-obstructive CAD (INOCA) or angina associated with non-obstructive CAD (ANOCA) may be caused by several mechanisms. INOCA has several main etiologies, including microvascular angina, vasospastic angina, mixed disease, and non-cardiac causes. INOCA is detected in up to 50% of patients with diagnosed or presumed angina. Most of these patients have microvascular and vasospastic angina, based on specific tests [7].

In addition to coronary microvascular dysfunction (CMVD), there is also micro-vascular vasospasm. This can be due to structural abnormalities or the coronary microcirculation incapacity to vasodilate appropriately. These abnormalities include decreased arteriole lumen with inward remodeling, capillary rarefaction, or even capillary compression due to myocardial hypertrophy or fibrosis [8].

Our past diagnostic tests were intended to identify ischemia caused by obstructive coronary artery disease. Previously, when a patient had a positive stress ECG test, but no obstructive coronary disease was found on angiography, we would dismiss the patient and assume that the ST segment depressions were “false positives”. However, the CANS study has shown that coronary microvascular dysfunction could cause these ECG modifications. During the cardiac autonomic nervous system (CANS) study, women with INOCA identified with coronary microvascular dysfunction through invasive evaluation were provided 24-hour ECG monitoring. The findings revealed that over one-third of these women had ST-segment depression, while no ECG modifications occurred in the comparator group [9].

To improve INOCA diagnosis, we can categorize methods into non-invasive and invasive approaches. Two primary non-invasive methods for diagnosing INOCA are PET and CMR. PET is beneficial because it measures rest and stress myocardial blood flow per gram of tissue, estimating myocardial flow reserve (MFR). Meanwhile, CMR imaging can detect semi-quantitative myocardial perfusion reserve index that may help determine coronary microvascular dysfunction (CMD), albeit in a few academic centers only [10]. Transthoracic Doppler echocardiography is a reliable method to visualize flow in the left anterior descending artery. Low coronary flow velocity reserve derived from this technique is a marker of coronary microvascular dysfunction. Adenosine is used as a pharmacologic stress agent.

Measuring myocardial blood flow (MBF) is feasible by utilizing phase-contrast cine cardiovascular magnetic resonance of the coronary sinus. This is because approximately 96% of the blood flow returning to the myocardium passes through it [11]. Therefore, the blood flow in the coronary sinus provides a reasonable estimate of the total MBF. To calculate the volume of blood flow per gram of myocardium, divide the coronary sinus blood flow by the weight of the myocardium. The MBF can be used to calculate CFR. Due to its radiation-free testing capability, CMR-derived CFR surpasses the limitations of PET-derived CFR. The potential of using CMR to assess CFR makes it a promising screening method. However, to determine the clinical significance of CMR-derived CFR, we require trials with large cohorts [11].

It is not possible for non-invasive stress testing methods to accurately detect microvascular spasms or coronary endothelial dysfunction. Even if a non-invasive stress test yields negative results, it cannot completely exclude coronary vasomotor dysfunction, particularly in patients displaying symptoms. In such cases, invasive coronary function testing may be required to properly evaluate coronary vascular function pathways. The current invasive approach for diagnosing INOCA involves coronary function testing and microvascular dysfunction diagnosis via pressure wire assessment by administering a vasodilator (adenosine) and acetylcholine intracoronary assessment for vasoreactivity [12].

The initial treatment for INOCA primarily consists of prescribing beta-blockers and calcium channel blockers as traditional therapy. However, no current head-to-head trials exist in subjects with INOCA.

Short-acting nitrates benefitted patients with vasospastic angina and stable anginal symptoms caused by epicardial obstructive coronary disease. However, they do not affect the microcirculation. Numerous patients with INOCA have a mixture of spasms and insufficient vasodilatory capacity, which is why nitrates are commonly used.

SGLT2 inhibitors may be a viable option as they have been proven to improve endothelial function through various mechanisms. The WARRIOR trial is currently investigating whether the use of optimal medical treatment (maximally tolerated dose of atorvastatin or rosuvastatin, lisinopril or losartan, and low-dose aspirin) could notably reduce major adverse cardiovascular events in women diagnosticated with INOCA [13]. The estimated study completion date is December 30, 2023.

## 3. The Role of Anatomical Assessment in Stable Coronary Syndromes

A comprehensive assessment of coronary anatomy, including the presence and location of atheroma. Coronary CT angiography in patients with anginal symptoms can determine whether a coronary artery has an abnormal course, most commonly between the aorta and pulmonary artery, and whether the coronaries are atheroma-free. If a patient is determined to have a significant atheroma burden, then OMT is indicated.

Medical therapy consists of two elements: disease-targeting therapies, such as aspirin, statins, and ACE inhibitors, based on the results of the HOPE and EUROPA studies [14, 15], and anti-anginal drugs. Anti-anginal drugs are commonly prescribed with beta-blockers to alleviate symptoms effectively. Numerous studies have confirmed medical therapy’s efficacy in treating and prognosis for patients with chronic coronary syndrome.

The Scottish Computed Tomography of the HEART (SCOT HEART) study included 4146 patients with stable chest pain randomly assigned to standard care alone or CTCA as their initial assessment [16]. After five years, the CTCA group had significantly lower death rates from CAD and nonfatal myocardial infarction [17]. Interestingly, despite similar overall revascularization rates, the improved results were attributed to adequate coronary artery disease identification and disease-modifying therapy implementation [18].

Based on the results of the ISCHEMIA trial, it may be possible to triage patients with stable chest pain using only CTCA to identify significant CAD and then proceed with OMT without additional testing. The ISCHEMIA trial was initially intended to enroll patients with stable angina with a moderate ischemia burden at baseline. However, over 10% of the participants had either mild ischemia or none. However, the trial found that undergoing early angiography and revascularization yielded no significant advantage in outcomes compared to OMT alone. This was measured by the primary composite endpoint, which included cardiovascular death, myocardial infarction, urgent hospitalization for unstable angina, or acute heart failure [19].

## 4. Coronary Physiology Assessment: Methods and Evidence

Examining the entire coronary tree with coronary physiological indexes is crucial to ensure an accurate diagnosis, which non-invasive methods can’t accomplish. Detecting coronary microvascular disease (CMVD) can be done by measuring coronary flow reserve (CFR) using techniques such as positron emission tomography, stress transthoracic echocardiography, and magnetic resonance imaging. However, these methods cannot accurately determine the degree of contribution of epicardial and microvascular disorders to the decrease in myocardial blood flow.

### 4.1 Invasive Methods for Assessing Microvascular Function

Coronary flow reserve is the ratio of the maximal or hyperemic flow down a coronary vessel to the resting flow. It can be assessed invasively through a Doppler-tipped guidewire with intracoronary or intravenous adenosine administration. Alternatively, has developed a thermodilution-derived method that estimates CFR and FFR simultaneously. CFR examines the entire coronary circulation, including the epicardial vessels and the microvasculature, and has become a diagnostic method for detecting micro-vascular dysfunction in individuals without obstructive epicardial coronary disease [20].

It is generally accepted that a typical CFR should exceed 2.0. A CFR value between 3 and 5 is considered normal for most patients. The CFR value will be significantly impacted if there is any hemodynamic disturbance [21]. The measurement of CFR includes resting flow, which results in more significant variability and less reliability in contrast to the measurement of microvascular resistance during hyperemia.

The index of microcirculatory resistance (IMR) is an assessment taken with a guidewire that allows for a quantitative appraisal of the minimum resistance in the microvascular system of a specific coronary artery. This value stays stable even when hemodynamic parameters fluctuate [22] and may indicate the extent of an infarct following primary PCI [23]. To properly assess the IMR, a specialized procedure must be followed. This involves using a coronary pressure-temperature sensor guidewire that is properly calibrated, along with a specific console, and administering adenosine or papaverine intracoronary to induce hyperemia. A FFR calculation is automatically documented, allowing the physician the capability to investigate both the epicardial vessel and the microvasculature simultaneously.

The normal range for IMR is less than 25. IMR provides better accuracy and less impact on hemodynamics than CFR and is similar to FFR [22]. IMR is unaffected by epicardial coronary stenosis unless there is a significant narrowing. In this scenario, IMR can be falsely elevated [24].

### 4.2 The Use of Quantitative Flow Ratio (QFR) to Evaluate Coronary Physiology

A quantitative flow ratio technique can rapidly estimate fractional flow reserve by combining three-dimensional quantitative coronary angiography and thrombolysis in myocardial infarction (TIMI) frame counting. Compared with FFR, QFR does not require invasive physiological measurements, pharmacological hyperemia induction, or additional cost. The FAVOR Pilot Study [25] and FAVOR II China Study [26] have indicated that QFR correlates well with wire-based physiological assessment. However, the study size in these studies was small. The diagnostic accuracy of QFR may be impacted in coronary arteries that have experienced a previous myocardial infarction [27]. This could be due to microcirculatory resistance.

### 4.3 The Use of Wire-Based Methods to Evaluate Coronary Physiology 

FFR is a hyperemic pressure wire assessment that measures the maximum blood flow in the heart’s area supplied by a narrowed coronary artery compared to the total blood flow in the same area if the artery was not narrowed. This is determined by calculating the ratio of the mean blood pressure downstream from the narrowed segment (Pd) to the mean pressure upstream from the segment (Pa) during peak blood flow and a minimum level of resistance [28]. To measure FFR, adenosine can be infused intravenously for 3 minutes at a dose of 140 µg/kg/min, or regadenoson can be given as a bolus intravenously at a dose of 400 µg. It can be administrated as an intracoronary bolus injection of adenosine (100 µg for the right coronary artery and 200 µg for the left coronary artery) or papaverine.

The instantaneous wave-free ratio (IFR) is an innovative measure of coronary stenosis severity that doesn’t rely on hyperemic pressure or potent pharmacological vasodilator agents like adenosine. Instead, it utilizes the unique properties of baseline coronary physiology and is taken during the wave-free period (WFP) of diastole when blood flow is at its highest. This approach amplifies the capacity to distinguish between stenosis severity and provides more accurate results than any other phase of the cardiac cycle [29].

The pressure wire is an effective tool for measuring downstream myocardial ischemia by analyzing the pressure drop across lesions in a vessel through FFR or IFR. Understanding vessel-specific and lesion-specific ischemia is crucial in determining the need for coronary stent implantation, as it is only beneficial in lesions that cause downstream ischemia. Numerous high-quality randomized trials, such as DEFER, FAME, and FAME2, have demonstrated the efficacy of this approach outside of acute ST elevation myocardial infarction (MI) [30, 31, 32].

The DEFER trial is the first randomized controlled trial to investigate the feasibility of Fractional Flow Reserve to guide percutaneous revascularization, enrolled 325 patients directed to elective interventional revascularization with stenosis deemed “significant” through angiography (with diameter stenosis of over 50%) and no documented ischemia. Before the intervention, FFR was measured.

Patients with hemodynamically insignificant lesions (FFR of over 0.75) were randomly assigned to either delayed PCI or interventional angioplasty. Patients with FFR of less than 0.75 lesions underwent PCI. Following PCI, angina-free patients were significantly more in FFR less than 0.75 before the PCI group. In those with FFR over 0.75, performing PCI had no positive effect on adverse cardiac events or angina relief compared to deferring the procedure. Follow-up data over 15 years indicated that all groups had the same death rate. Still, patients with normal FFR in the performing group had a significantly higher MI rate than those in the deferred group [33].

The DEFER approach suggests that PCI is only beneficial for lesions that cause downstream ischemia, while non-ischemic lesions are better treated with OMT alone. Trials such as DEFINE-FLAIR and IFR-SWEDEHEART revealed that non-ischemic lesions can be safely treated with deferred revascularization using IFR or FFR [34].

The FAME study (Fractional Flow Reserve versus Angiography for Guiding Percutaneous Coronary Intervention) involved enrolling over 1000 patients with multivessel CAD; participants were randomly selected to either undergo angiographically-guided revascularization of all eligible lesions or FFR-guided revascularization of the lesions with FFR less than 0.8 [31]. The FFR-guided group showed a significantly lower primary composite endpoint of death, MI, and repeat revascularization. The differences in rates of MI (8.7% vs. 5.7%, relative risk 0.66, *p* = 0.07) and repeat revascularization (9.5% vs. 6.5%, *p* = 0.08) were more pronounced than those for mortality (3.0% vs. 1.8%, *p* = 0.19). Remarkably, improved outcomes in the FFR-guided group were achieved despite fewer stents being placed per patient (2.7 ± 1.2 vs. 1.9 ± 1.3, *p* < 0.001) and lower procedure-related costs. This occurred because over one in three (angiographically “significant”) lesions in the FFR group were hemodynamically normal and left unstented.

The FAME2 study aimed to determine whether patients with functionally significant stenosis (FFR ≤0.80) suitable for PCI would benefit more from PCI with OMT or OMT alone. Unfortunately, the study had to be stopped prematurely after 1220 patients were enrolled since the PCI group showed substantially lower rates of composite primary endpoints: myocardial infarction, need for urgent revascularization, and death (4.3% vs. 12.7%). The primary cause of this disparity was the PCI group’s lower incidence of urgent revascularization (1.6% vs. 11.1%, 95% CI 0.06–0.30) [32].

The FAME 3 investigation (Fractional Flow Reserve–Guided PCI as Compared with Coronary Bypass Surgery) concentrated on patients diagnosed with three-vessel coronary artery disease through angiography. The study found that FFR-guided PCI was less effective than CABG in preventing a combination of death, MI, stroke, or repeat revascularization within a year [35].

After conducting these trials, it can be concluded that it is better to medically treat non-ischemic stenting lesions instead of stenting them, as shown in DEFER [33]. Deferring non-ischemic lesions based on FFR or IFR results in a positive medium-term outcome with low ischemic events [34]. Patients with pressure wire-positive lesions who undergo stenting have a lower event rate, primarily driven by urgent revascularization, than those who receive OMT alone, as shown in FAME2 [32].

Multivessel PCI guided by FFR yields improved clinical outcomes with lower rates of myocardial infarction, the need for repeat revascularization, and death compared to angiographically guided PCI. Using fewer scaffolds in fewer vessels has proven to be cost-effective and highly effective in optimizing PCI planning, as shown in FAME [32].

### 4.4 Limitations of FFR-Guided PCI

Ideally, post-PCI FFR values, corresponding to an FFR of 0.90 or higher, are associated with better outcomes, such as lower MACE and angina. Even with satisfactory results from angiography, up to 30–65% of patients may have suboptimal post-PCI FFR values, while up to 20% may have poor FFR values (FFR of 0.80 or lower). Various factors, such as diffuse CAD without focal lesions, residual lesions inappropriate for PCI, stent malposition or suboptimal expansion, edge dissection, and plaque protrusion, can affect Post-PCI FFR values [2]. Treatment usually involves post-dilation or further stenting, usually using intracoronary imaging techniques, such as IVUS and OCT. The factors influencing the post-PCI FFR may also contribute to future atherosclerosis and target vessel failure, especially in patients with diffuse coronary disease and residual disease. Also, the source of abnormal or damaged post-PCI FFR values usually lies outside the stent. There is also a possibility that patients at higher risk for MACE or target vessel failure may also have lower post-PCI FFR values, whether or not a causal relationship exists.

### 4.5 Computed Tomography-Based FFR (FFR CT), a Real Option? 

A reliable and proven technique called FFR CT can effectively model FFR in the major coronary vessels using computed tomography [36]. This technique can evaluate atheroma magnitude, pattern, and presence, along with vessel-specific ischemia. This involves creating an anatomical model of the arteries and a physiological model of the circulation process. Resting coronary flow is calculated based on myocardial mass, the maximum hyperemia is estimated by considering the expected reduction in resistance with adenosine injection and the FFR CT is then measured using supercomputers and computational fluid dynamics methods.

FFR CT provides additional anatomical information within physiological assessment, lowering the number of invasive coronary angiography exams and the need for invasive FFR measurement, a cost-efficient method, and noninferiority compared with invasive FFR. Several studies confirm the reliability of this noninvasive assessment for stable angina patients, like PACIFIC, ADVANCE, and TARGET trials [37, 38, 39].

When it comes to non-invasive tests, the diagnostic performance of this particular one is quite intriguing. Driessen *et al*. [37] conducted a study comparing the assessment of coronary ischemia using FFR CT with other noninvasive stress tests. According to the study, FFR CT had a higher area under the receiver-operating characteristic curve (AUC) for identifying lesions that cause ischemia than coronary CTA and SPECT. Concerning the results, FFR CT performed better than PET on a per-vessel basis with an AUC of 0.87 (*p* < 0.01) but not on a per-patient basis with an AUC of 0.91 (*p* = 0.56) [37].

The results are a post hoc sub-analysis of the PACIFIC investigation. FFR CT rated a substantial number (17%) of vessels as non-valuable and, as a result, excluded them from the primary analysis. The drop-out rate will decrease as CT equipment and software are improved.

The effectiveness of FFR CT in guiding management was again proven through the ADVANCE registry. This registry comprised 5083 patients with suspected CAD diagnosed with atherosclerosis due to over 30% stenosis on CTCA [38]. In 66.9% of cases, management plans were revised due to FFR CT results availability. One note-worthy finding was the significantly lower rate of occurrence of cardiac events in those with a negative FFR CT (43 major events in patients with FFR CT ≤0.80 compared to only 12 in those with FFR CT >0.80), providing reassuring evidence for those with coronary disease.

The TARGET trial, the first randomized FFR CT-guided management trial for patients with stable coronary disease, was published in Circulation [39]. The study was conducted in six medical centers in China, involving 1216 patients with stable coronary artery disease and intermediate stenosis of 30% to 90% on coronary computed tomographic angiography. These patients were randomly assigned to either an on-site FFR CT care using machine learning or standard care. The investigation’s main purpose was to determine the proportion of patients who underwent invasive coronary angiography without obstructive coronary artery disease or intervention within 90 days. Secondary measurements included significant adverse cardiovascular events, quality of life, angina symptoms, and medical expenditures at one year. Both groups had similar baseline characteristics, with 72.4% (881/1216) experiencing typical or atypical anginal symptoms.

In the FFR CT care group, 69.2% (421/608) of patients underwent invasive coronary angiography, compared to 79.4% (483/608) in the standard care group. The FFR CT care group saw a significant reduction in the percentage of patients who underwent invasive coronary angiography free of obstructive coronary artery disease or with obstructive disease not undergoing intervention compared to standard care (28.3% [119/421] vs. 46.2% [223/483]; *p* < 0.001). While more patients in the FFR CT care group underwent revascularization than in the standard care group (49.7% [302/608] vs. 42.8% [260/608]; *p* = 0.02), there was no significant difference in major adverse cardiovascular events at one year (hazard ratio, 0.88 [95% CI, 0.59–1.30]). Both cohorts showed similar improvements in quality of life and symptoms during follow-up, and there was a trend towards lower costs in the FFR CT care group [39].

One area that still needs improvement is the offline analysis of CCTA image data sets. Sending the data for post-processing can take 1 to 4 hours and can be costly. Moreover, the accuracy of the analysis is heavily influenced by the image quality, which can be affected by factors such as motion artifact, severe calcification, and stenting, all of which can decrease the data analyzability.

The TARGET trial reveals that on-site FFR CT decreased the proportion of patients with stable CAD who required ICA and did not need a procedure within 90 days. However, it also resulted in a significant increase in revascularization, lacking any improvement in health outcomes, quality of life, or lower primary adverse cardiovascular outcomes.

## 5. Combining Anatomy and Physiology Assessments during the Evaluation Stage: A Promising Strategy?

Considering the link between ischemic burden and outcome, it has been demonstrated that using pressure wire with angiography during PCI contributes substantially better results than only angiographic assessment. FFR CT can aid in assessing and treating patients with positive clinical outcomes while decreasing the need for invasive angiography. Due to this, it is reasonable to assume that routinely investigating the anatomy and physiology of all epicardial coronary arteries would lead to better diagnostic outcomes than relying exclusively on invasive angiography (either CTCA or traditional). Additionally, considering the economic analyses conducted by FAME for invasive procedures and TARGET for noninvasive procedures, it is plausible to assume that implementing such a strategy could potentially result in cost-effectiveness [33, 39].

Two randomized trials have been conducted to test the proposed concept. The first trial RIPCORD2 [40] involved invasive angiography and pressure wire evaluation, while the second trial FORECAST utilized FFR CT [41].

The RIPCORD2 trial enrolled 1100 patients undergoing invasive coronary angiography to study stable angina or non-ST elevation MI. In order to participate, individuals needed to have a coronary vessel with at least one stenosis of 30% or more that could be treated by interventional revascularization. Participants were randomly assigned to receive either evaluation and management based solely on angiographic findings or angiographic interpretation in combination with comprehensive FFR measurement in all epicardial vessels. The patients who underwent angiographic assessment plus FFR had an average of four blood vessels examined using FFR. Although this method resulted in more prolonged cases and higher contrast usage, there was no significant difference in the primary outcomes of overall hospital expenses, quality of life, and angina symptoms after one year. The two groups displayed comparable all-cause mortality rates, non-fatal stroke, non-fatal myocardial infarction, and emergency revascularization.

The experimental strategy has effectively reduced the number of patients requiring additional tests. Precisely, only 1.8% of patients needed more tests compared to 14.7% in the control group (*p* < 0.00001) [40].

In the FORECAST trial, 1400 patients with stable chest pain were randomly assigned to either initial testing with CTCA and selective FFR CT or conventional treatment. The study found no significant variation in the average total cardiac expenses after nine months between both groups. It did not reveal any differences in clinical outcomes, including quality of life, angina occurrence, or any significant adverse cardiac and cerebrovascular events. However, the FFR CT arm had lower rates of invasive coronary angiograms and a reduced proportion of angiograms showing no obstructive epicardial lesion. These findings suggest that CTCA with selective FFR CT is a viable alternative to standard clinical care in patients with stable angina, with the added benefit of reducing invasive procedures [41].

## 6. Exploring the Patterns of Coronary Atherosclerosis and Selecting the Best Course of Treatment

Anatomy and physiology are two significant fields studied together. However, there is a wide gap regarding the lesions’ significance. As part of this assessment, the pattern of CAD is evaluated as either focal or diffuse. Patients with focal angiographic disease establish a diffuse pattern of pressure loss along the coronary vessel. On the contrary, patients with diffuse angiographic CAD can manifest focal pressure loss. However, it is essential to understand the implications of the distinct patterns to develop tailored treatment plans for each patient.

The pullback pressure gradient (PPG) is a new metric that assesses the patterns associated with CAD using FFR pullbacks. This measurement incorporates the magnitude and extent of pressure losses, giving a metric that varies from 0 to 1. Values near 0 correspond to diffuse CAD, whereas values near 1 indicate focal CAD [42].

For the calculation of the PPG index and for characterizing the functional pattern of CAD, a combination of two parameters was used: (1) a maximal PPG over 20 mm, which represents the magnitude of the decrease in FFR, and (2) the length of epicardial coronary segments with the decrease in FFR. This formula can be used to calculate PPG:

$$\begin{array}{c} \text{PPG index }=\frac{\left\{\frac{\text{ Max PPG 20 mm }}{\Delta \,\text{ FFR vessel }}+\left(1-\frac{\text{ Length with functional disease }\,\left(\mathrm{mm}\right)}{\text{ Total vessel length }\,\left(\mathrm{mm}\right)}\right)\right\}}{2} \end{array}$$

Max PPG was established as the maximum pressure gradient over 20 mm, and delta FFR vessel as the difference between the FFRs measured at the ostium of the vessel and the most distal anatomical site. Motorized pullback pressure tracing determines both the length of functional disease and the length of the total vessel. Functional CAD was defined as millimeters, with an FFR drop ≥0.0015/mm [42].

**Fig. 1.**
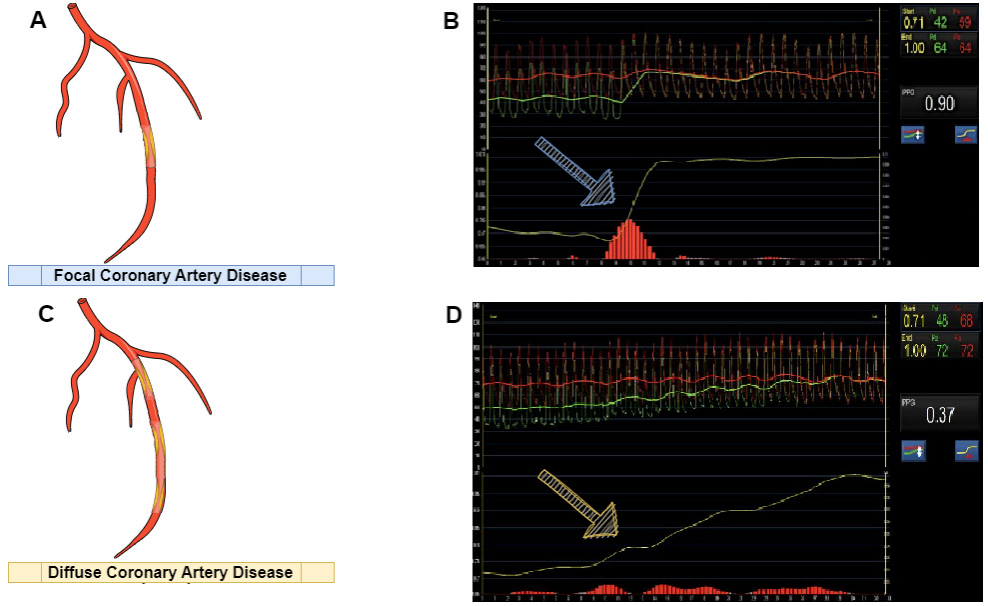
**Types of coronary artery disease and physiological assessment**. (A) Anatomical view of focal artery disease. (B) Physiological assessment of focal artery disease. (C) Anatomical view of diffuse artery disease. (D) Physiological evaluation of diffuse artery disease; blue arrow: appearance of a sudden and wide change in the pressure curve is a common characteristic of focal disease; yellow arrow: appearance of a discrete shift in the pressure curve is a common characteristic of diffuse disease.

Collet *et al*. [42] assessed FFR pullbacks in 85 vessels and reported the PPG index, with average values of 0.37 ± 0.07 (28 vessels) suggesting diffuse disease, 0.77 ± 0.08 (29 vessels) indicating focal disease, and 0.57 ± 0.05 (28 vessels) representing mixed disease. The results suggest that lower PPG values are associated with diffuse disease, while higher PPG values are related to focal illness (Fig. 1).

Unlike conventional angiography, motorized FFR pullbacks reclassified 36% of the vessel disease patterns [42].

A revascularization strategy is determined by the type of coronary atherosclerosis (focal or diffuse). While FFR-guided PCI is clinically beneficial, one-third of patients retain suboptimal post-PCI FFR associated with significant adverse events, making PCI in diffuse CAD questionable. Several randomized and observational studies have shown that a low FFR post-PCI is associated with adverse clinical outcomes [43, 44] and a high risk of target vessel revascularization, myocardial infarction, as well cardiac death [44, 45]. A diffusely diseased coronary vessel is assumed to require longer stents when PCI is performed. Interestingly, Baranauskas *et al*. [46] conducted a study and found that the FFR results post-PCI were suboptimal in most patients treated with extended drug-eluting stent (DES) and were particularly poor when the stent was longer than 50 mm.

Current strategies focus on treating diffuse coronary disease conservatively using medical therapy or bypass surgery since PCI is not the best choice. Even coronary artery bypass grafting patients have a poor prognosis when suffering from diffuse disease.

Shiono *et al*. [47] examined the effect of functional focal coronary artery disease versus diffuse coronary artery disease on the patency of bypass grafts. They studied 89 patients subjected to measure the pressures within the guidewire pullback in the left anterior descending (LAD) artery before CABG using the internal mammary artery (IMA). Pressure guidewire pullback data classified the LAD lesions as functional focal disease (abrupt pressure step-up; n = 58) or functional diffuse disease (gradual pressure increase; n = 31). In a follow-up CT angiography within one year following CABG, it was observed that diffuse disease in the LAD had been associated with a higher rate of left internal mammary artery graft occlusion compared with focal disease (26% vs. 7%, *p* = 0.021) [47].

It has been suggested by Ellouze *et al*. [48] that coronary endarterectomy may be an alternative surgical treatment option for patients with diffuse coronary artery disease not suitable for coronary bypass surgery alone. Between January 2015 and January 2018, 147 consecutive patients completed 154 adjunctive CE (coronary endarterectomy) interventions for advanced CAD. A study group of 32 consecutive patients who had computed tomography angiography after June 2016 underwent CTA for evaluating graft and coronary patency. CE was performed on 102 patients in the right coronary artery, 22 in the left anterior descending artery, and 17 in the circumflex artery. A procedural myocardial infarction occurred in seven patients (5%), while no perioperative deaths occurred. A CT scan was conducted three months after the surgery. The mean patency of endarterectomies, coronary arteries, and bypass grafts was 90% and 88%, respectively. The LAD arterial grafts were all patent. Based on the study’s results, the survival rate and the freedom from major adverse cardiovascular events were 95% ± 2% and 95% ± 6%, respectively. After three years of follow-up, patients had a patency rate of 100% [48].

**Fig. 2.**
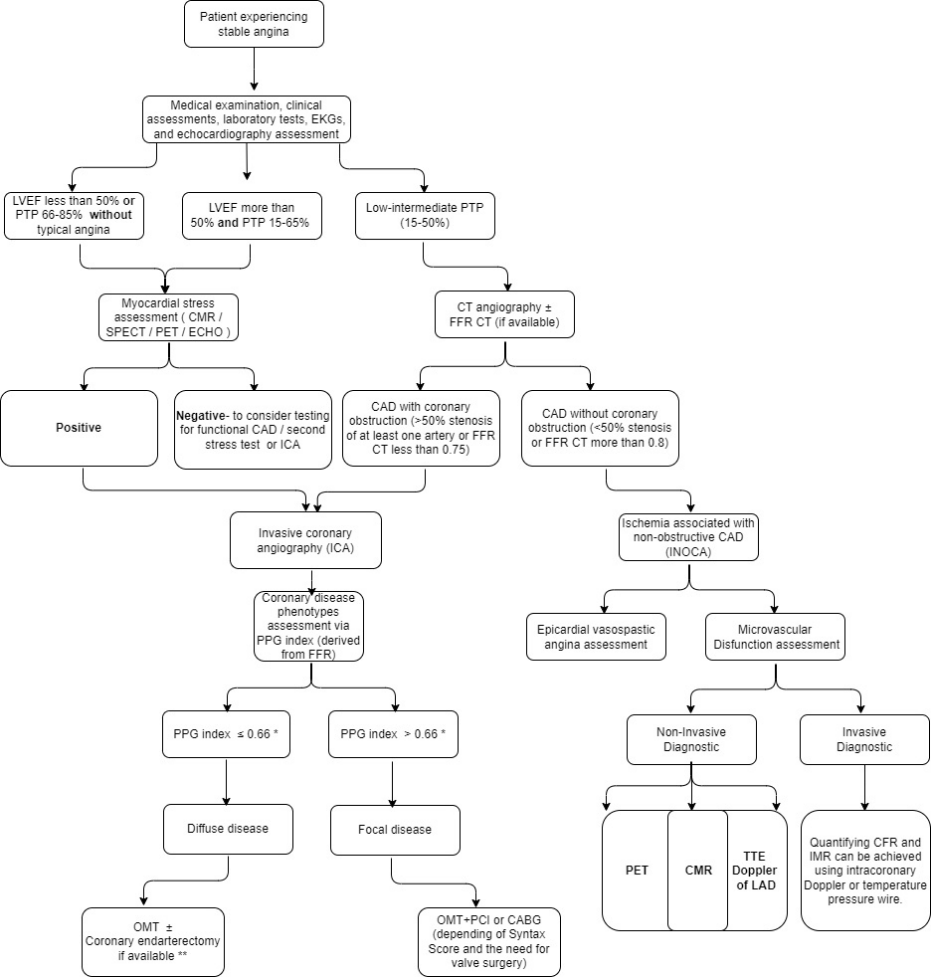
**Proposed flowchart for stable angina assessment and treatment methods**. * The proposed cut-off value for the PPG index is still under consideration. ** This is only a proposal for treating coronary diffuse disease. LVEF, left ventricular ejection fraction; PTP, pre-test probability; CMR, cardiovascular magnetic resonance; SPECT, single photon emission computed tomography; PET, positron emission tomography; ECHO, echocardiogram; FFR, fractional flow reserve; PPG, pullback pressure gradient; CT, computed tomography; TTE, transthoracic echocardiogram; CFR, coronary flow reserve; IMR, index of microcirculatory resistance; OMT, optimal medical therapy; PCI, percutaneous coronary intervention; CABG, coronary artery bypass graft surgery; EKGs, electrocardiograms; CAD, coronary artery disease; LAD, left anterior descending .

We propose a flowchart for stable angina assessment and treatment methods (Fig. 2) based on the medical examination, electrocardiogram (EKG) and echocardiography assessment, LVEF function, myocardial stress tests, FFR CT, ICA and PPG index.

A considerable study named PPG Global Registry (NCT04789317) is underway investigating the clinical impact of focal and diffuse CAD in a large population to gather more evidence for adequate treatment.

## 7. Conclusions

Modern interventional cardiology remains challenged by serial stenosis evaluation. It may be possible to treat these demanding lesions with invasive coronary physiology. This includes stenosis evaluation by FFR and disease diffuseness by PPG. To diagnose and treat angina, the pullback gradient index may help develop an effective treatment plan to manage angina symptoms. It provides a means to refine the appropriateness criteria for PCI to avoid treating patients with diffuse disease or at least increase awareness of diffuse disease and the fact that patients with diffuse disease are unlikely to improve.

In addition to improving patient selection for revascularization, the PPG may be useful for identifying patients for whom PCI will provide superior results before treatment is initiated. Further randomized clinical trials are required to explore the validity of a PPG-guided PCI strategy.

There is an ongoing study investigating the clinical impact of focal and diffuse CAD in a large population in the PPG Global registry (NCT04789317), with a projected completion date of 31 December 2025.
